# Classification of the glioma grading using radiomics analysis

**DOI:** 10.7717/peerj.5982

**Published:** 2018-11-22

**Authors:** Hwan-ho Cho, Seung-hak Lee, Jonghoon Kim, Hyunjin Park

**Affiliations:** 1 Department of Electronic and Computer Engineering, Sungkyunkwan University, Suwon, Korea; 2 Center for Neuroscience Imaging Research, Institute for Basic Science, Suwon, Korea; 3 School of Electronic and Electrical Engineering, Sungkyunkwan University, Suwon, Korea

**Keywords:** Machine learning, Multi-modal imaging, Radiomics, Glioma grading

## Abstract

**Background:**

Grading of gliomas is critical information related to prognosis and survival. We aimed to apply a radiomics approach using various machine learning classifiers to determine the glioma grading.

**Methods:**

We considered 285 (high grade *n* = 210, low grade *n* = 75) cases obtained from the Brain Tumor Segmentation 2017 Challenge. Manual annotations of enhancing tumors, non-enhancing tumors, necrosis, and edema were provided by the database. Each case was multi-modal with T1-weighted, T1-contrast enhanced, T2-weighted, and FLAIR images. A five-fold cross validation was adopted to separate the training and test data. A total of 468 radiomics features were calculated for three types of regions of interest. The minimum redundancy maximum relevance algorithm was used to select features useful for classifying glioma grades in the training cohort. The selected features were used to build three classifier models of logistics, support vector machines, and random forest classifiers. The classification performance of the models was measured in the training cohort using accuracy, sensitivity, specificity, and area under the curve (AUC) of the receiver operating characteristic curve. The trained classifier models were applied to the test cohort.

**Results:**

Five significant features were selected for the machine learning classifiers and the three classifiers showed an average AUC of 0.9400 for training cohorts and 0.9030 (logistic regression 0.9010, support vector machine 0.8866, and random forest 0.9213) for test cohorts.

**Discussion:**

Glioma grading could be accurately determined using machine learning and feature selection techniques in conjunction with a radiomics approach. The results of our study might contribute to high-throughput computer aided diagnosis system for gliomas.

## Introduction

Gliomas are primary brain tumors arising from glial cells. The grades of gliomas have been determined based on histology according to the World Health Organization standard (Louis et al., 2007). Recently, revised criteria have been introduced that consider genetic factors such as isocitrate dehydrogenase mutation and 1p/19q codeletion (Louis et al., 2016). The grading of gliomas is critical information related to prognosis and survival (Wu et al., 2010; Louis et al., 2016). A scheme that dichotomizes the graded gliomas into high-grade gliomas (HGG) and low-grade gliomas (LGG) has been widely adopted. It is important to differentiate HGG from LGG for assessing tumor progression and therapy planning (Louis et al., 2007). An experienced observer can differentiate between the two grades well based on tumor enhancement, but a computer algorithm might match the performance of the human expert with increased speed. More importantly, the computer algorithm might contribute to developing high-throughput computer aided diagnosis system.

An algorithm known as radiomics has recently emerged as a powerful methodology to quantify the characteristics of tumors in a non-invasive manner (Yip & Aerts, 2016). Many studies have demonstrated that distinct tumor types in many organs can be quantified by radiomics analysis and the results of the radiomics can be used as imaging biomarkers for supporting clinical decision making (Zacharaki et al., 2009; Aerts et al., 2014; Kickingereder et al., 2016; Li et al., 2016; Bowen et al., 2017). Radiomics can also reveal novel characteristics of brain tumors, as demonstrated by a recent study (Zhou et al., 2017b). Many studies predicted the chemotherapeutic response and survival of patients with glioblastoma using a large number of imaging features based on MR imaging (Itakura et al., 2015; Cui et al., 2016; Kickingereder et al., 2016; Prasanna et al., 2016; Lao et al., 2017; Zhou et al., 2017a). Other studies have predicted prognosis using features obtained from functional imaging (Ryu et al., 2014; Lee et al., 2016). Recently, radiomics has been combined with genomics to leverage two distinct types of information to better study various tumor types (Gutman et al., 2015; Li et al., 2016; Beig et al., 2018; Zinn et al., 2018). The new approach is referred to as radiogenomics and has to potential to reveal novel findings combining two distinct high dimensional information of gene and imaging information. Many existing brain tumor studies related to radiomics mainly focused on glioblastoma which is the most aggressive glioma and considered a limited number of imaging modalities (Ryu et al., 2014; Itakura et al., 2015; Cui et al., 2016; Lee et al., 2016). Multi-modal data are high-dimensional by nature and thus handling them properly requires carefully chosen machine learning approaches. However, existing literature of radiomics using multi-modal data and various machine learning approaches is relatively scarce.

**Figure 1:**
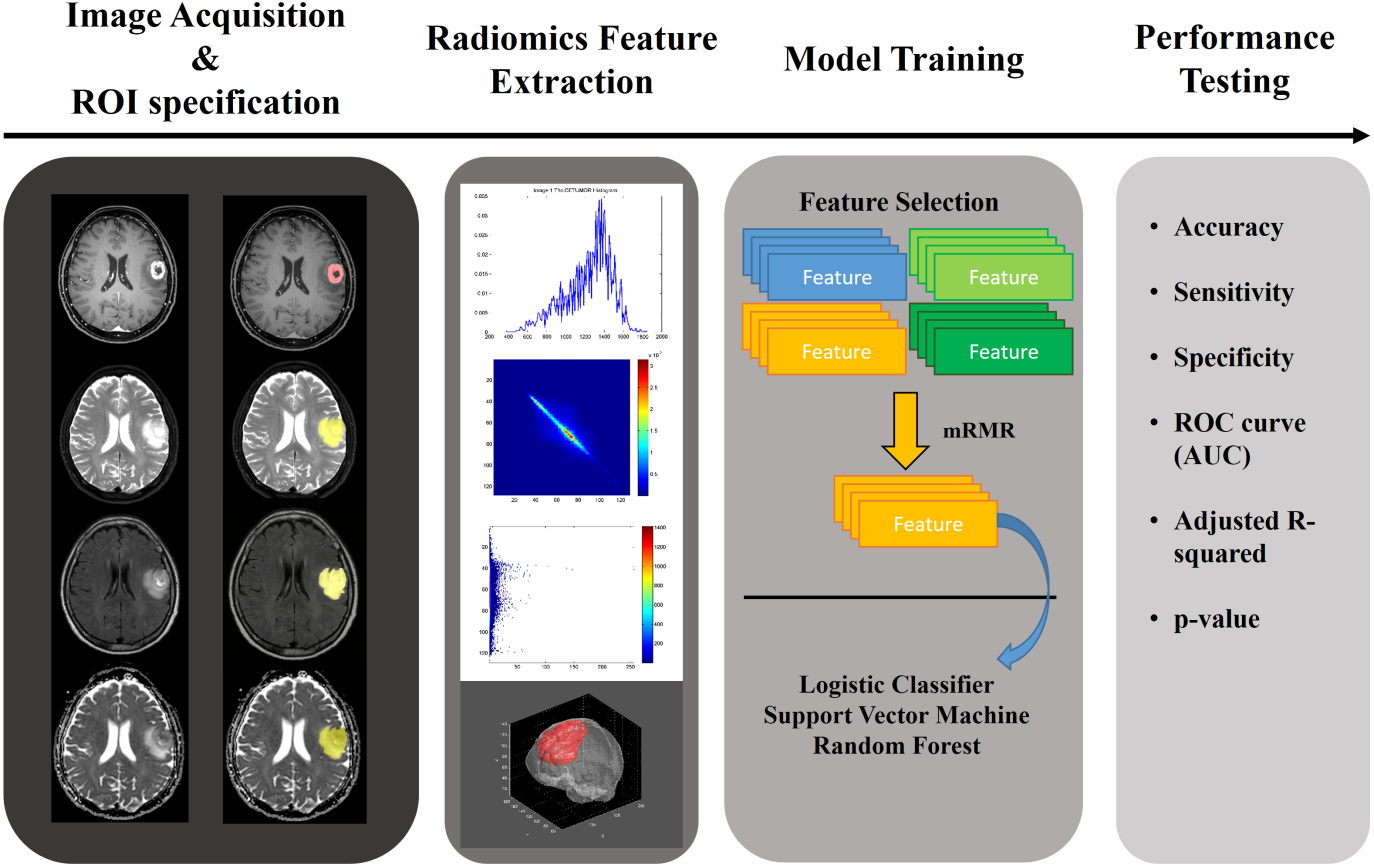
Overall workflow of the study.

In this paper, we applied a radiomics approach combined with various machine learning approaches to multi-modal imaging of glioma patients to study whether the grade of glioma can be determined noninvasively. The aim of this study was to quantify glioma with a radiomics approach and to use the results to classify the gliomas as HGG or LGG. We used annotated multi-modal MRI imaging data from a research database (Menze et al., 2015; Bakas et al., 2017a; Bakas et al., 2017b; Bakas et al., 2017c). A total of 468 quantitative radiomics were computed for four MRI modalities and three regions of interest (ROIs). Significant features were selected using relevance and redundancy criteria. We aimed to demonstrate the effectiveness of these features on the classification of each glioma’s histopathological grade using three machine learning classifiers. The overall workflow of this paper is shown in Fig. 1.

## Materials and Methods

### Patients and imaging

The institutional review broad (IRB) of Sungkyunkwan University approved our study (IRB# 2015-09-007). Consent was waived for this retrospective study. Our study was performed in full accordance with local IRB guidelines. We considered data from the MICCAI Brain Tumor Segmentation 2017 Challenge (BraTS 2017) (Menze et al., 2015; Bakas et al., 2017a; Bakas et al., 2017b; Bakas et al., 2017c). This dataset was derived from pre-operative baseline scans from two variants of the Cancer Imaging Archive (TCIA) (Clark et al., 2013), the TCIA-glioblastoma (GBM) and TCIA-LGG collections (Pedano et al., 2016; Scarpace et al., 2016). Each is a multi-institutional data mix from eight and five institutions, respectively. Detailed patient and scanner information can be found in the data citation (Bakas et al., 2017a; Bakas et al., 2017c; Bakas et al., 2017b). We considered 210 HGG and 75 LGG patients. HGG included glioblastoma multi-forme (GBM) and LGG included astrocytomas, oligodendroglioma, and oligoastrocytomas (Pedano et al., 2016; Scarpace et al., 2016; Bakas et al., 2017a).

Each patient had pre-operative images in four modalities (T1, T1-contrast enhanced, T2, FLAIR). All images were preprocessed using the FMRIB Software Library (FSL). Each image was registered onto the common space (Rohlfing et al., 2010) and interpolated to a 1 × 1 × 1 mm isotropic voxel grid. In addition, manual segmentations of enhancing tumors, non-enhancing tumors, necrosis, and edema in each image were also provided by the challenge organizers (Bakas et al., 2017a; Bakas et al., 2017c; Bakas et al., 2017b). Manual segmentation was performed using a semi-automatic method with expert confirmation (Bakas et al., 2017a). These specific preprocessing choices were made by the BraTS organizational committee (Menze et al., 2015; Bakas et al., 2017a). Our study was a single source study (just from the BraTS database) and thus we adopted a five-fold cross validation to separate the training and test cohorts to reduce overfitting. Each fold had a similar ratio of HGG and LGG. The ratio of HGG and LGG was maintained between the training and test sets. Table 1 contains institutional information for all patients.

**Table 1:** Institutional information of patients (Bakas et al., 2017a).

Collection	Institutions	TCGA ID
TCGA-GBM	Henry Ford Hospital, Detroit, MI	TCGA-06
	CWRU School of Medicine, Cleveland, OH	TCGA-19
	University of California, San Francisco, CA	TCGA-08
	Emory University, Atlanta, GA	TCGA-14
	MD Anderson Cancer Center, Houston, TX	TCGA-02
	Duke University School of Medicine, Durham, NC	TCGA-12
	Thomas Jefferson University, Philadelphia, PA	TCGA-76
	Fondazione IRCCSInstituto Neuroligico C. Besta, Milan, Italy	TCGA-27
TCGA-LGG	St Joseph Hospital/Medical Center, Phoenix, AZ	TCGA-HT
	Henry Ford Hospital, Detroit, MI	TCGA-DU
	Case Western Reserve University, Cleveland, OH	TCGA-FG
	Thomas Jefferson University, Philadelphia, PA	TCGA-CS
	University of North Carolina, Chapel Hill, NC	TCGA-EZ

**Notes.**

TCGA The Tumor Genome Atlas

### Tumor regions of interest

**Figure 2:**
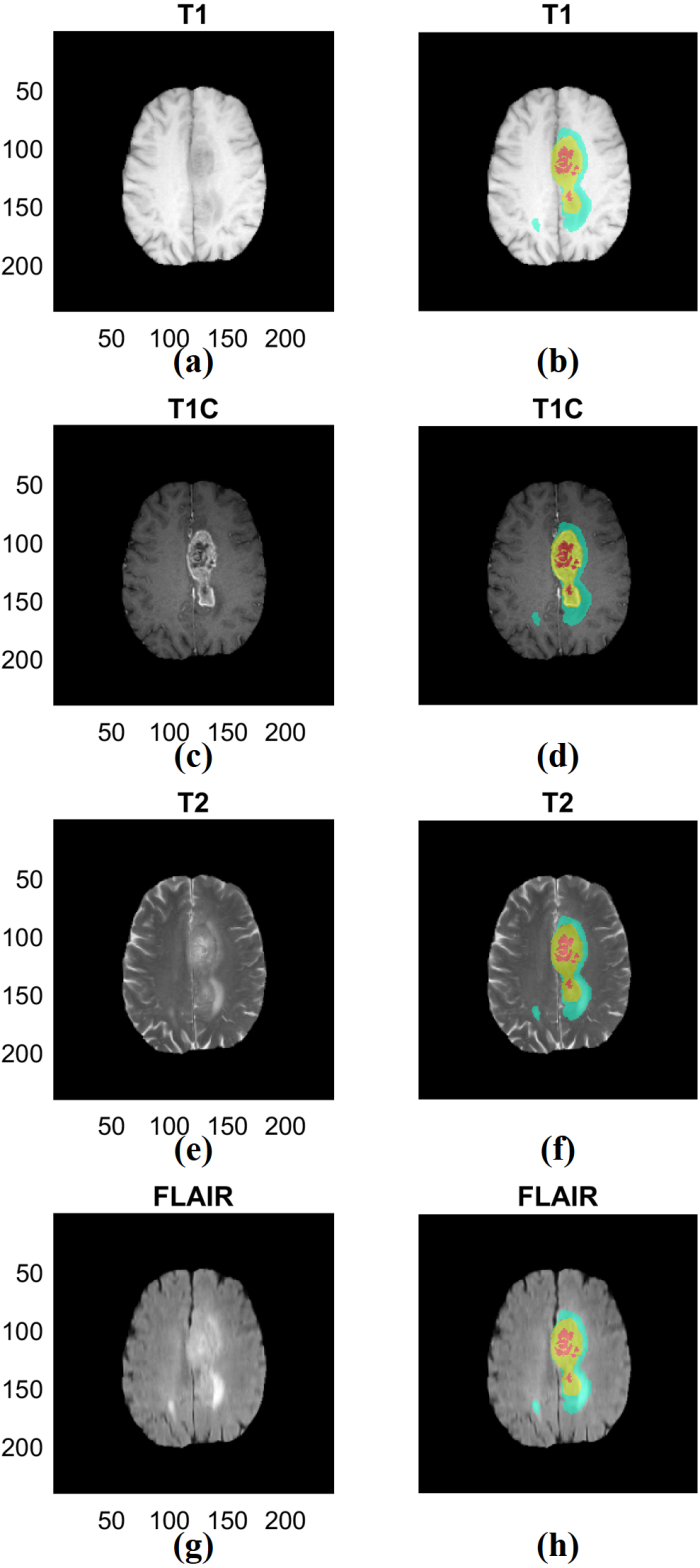
Examples of three types of ROIs used in our study. (A) T1 data. (B) ROI associated with T1. (C) T1C data. (D) ROI associated with T1C. (E) T2 data. (F) ROI associated with T2. (G) FLAIR data. (H) ROI associated with FLAIR. The left column (A) (C) (E) (G) shows different imaging modalities. The right column (B) (D) (F) (H) shows associated ROIs. The ROIs were specified in 3D but 2D representative examples are given. ROIs are visualized in the right column. Red indicates non-enhancing tumor and necrosis (ROI type I), yellow indicates enhancing tumor (ROI type II) and blue indicates edema (ROI type III) in the right column. T1; T1-weighted image, T2; T1C; T1-contrast enhanced; T2-weighted image, FLAIR; Fluid-Attenuated Inversion Recovery.

We combined the three manual segmentation results provided by BraTS into ROIs to extract multi-regional radiomics features. We intended to obtain information from multiple tissue types rather than single tissue type (Zacharaki et al., 2009). The first region (ROI type I) was created by merging the non-enhancing tumor and necrotic region and the second region (ROI type II) was created by adding the tumor region with enhancement to the first region. The third region (ROI type III) combined the second region with the area of edema. The first region is the smallest region and the third region is the largest region inclusive of multiple compartments. Representative ROIs are shown in Fig. 2.

### Radiomics features

To compute high-dimensional imaging information needed for the radiomics approach, imaging features were calculated using all three ROIs in four modalities from 3D volume. The features were computed using a combination of open source code (Van Griethuysen et al., 2017) and in-house generated computer code implemented in MATLAB (Mathworks Inc. Natick, MA, USA). For most features, we used the open source software PyRadiomics so that the results could be easily reproduced. For the features not available in PyRadiomics, we used our in-house MATLAB code which is provided as a supplement material. We computed a total of 468 radiomics features per patient. We computed 24 shape-based (eight for each ROI), 228 histogram-based, and 216 texture-based (192 gray-level co-occurrence matrix [GLCM] based and 24 intensity size-zone [ISZ] matrix-based) features quantifying different characteristics of the tumor (Haralick, Shanmugam & Dinstein, 1973; Tixier et al., 2011; Davnall et al., 2012; Grove et al., 2015). The histogram-based features were computed from 128 bin histogram computed over the whole intensity range. For the GLCM features, we binned intensities with 128 bins. A total of 26 matrices corresponding to 26 3D directions with offset one were computed and then averaged to yield one matrix. The averaged matrix was used to compute GLCM features. For the ISZM features, we constructed a 128 × 256 matrix where the first dimension was binned intensity and the second dimension was size. The size was not quantized and if a blob was larger than 256 voxels, it was considered as a blob with size 256. We considered six neighbors (four in-plane and two out-of-plane ones) for defining the size of the blob. More details can be found in the supplement.

### Feature selection

Feature values of the training cohort were normalized to *z*-scores for each feature across subjects. Different radiomics features have different units and range. Some features were designed to fall between 0 and 1, while others have a very large range. All the features were subjected to the feature selection procedure. Without feature normalization, some features might be assigned a larger weight, while others might be assigned a lower weight depending on the distribution of feature values during the feature selection. Thus, we applied *z*-score normalization to the feature values, making the range of each feature relatively uniform. A similar approach can be found in another work (Kickingereder et al., 2016). We selected image features which could distinguish between HGG and LGG using the minimum redundancy maximum relevance (mRMR) algorithm (Peng, Long & Ding, 2005). The mRMR is described with three equations. The first Eq. (1) searches for a set of features that maximizes the relevance (*D*), where *S* is the total feature set, *c* is the grade of glioma, *x*_*i*_ is the individual feature, and *I* is the information measure. The second Eq. (2) searches for a set of features that minimizes redundancy (*R*) among the selected features, where *x*_*i*_, *x*_*j*_ are the individual features. Equation (2) suppresses overlapping information among the selected features. The previous steps are combined into the third Eq. (3), where the mRMR algorithm identifies the optimal set of features that encourage maximum relevance and minimum redundancy. We performed mRMR with mutual information as the information measure and selected the top five features. We chose to select five features because we sought a compact model to distinguish between HGG and LGG images.

(1) \begin{eqnarray*}& & \max \nolimits D \left( S,c \right) ,D= \frac{1}{ \left\vert S \right\vert } \sum _{{x}_{i}\in S}I({x}_{i};c).\end{eqnarray*}

(2) \begin{eqnarray*}& & \min \nolimits R \left( S \right) ,R= \frac{1}{{ \left\vert S \right\vert }^{2}} \sum _{{x}_{i},{x}_{j}\in S}I({x}_{i},{x}_{j}).\end{eqnarray*}

(3) \begin{eqnarray*}& & \max \nolimits \Phi \left( D,R \right) ,\Phi =D-R.\end{eqnarray*}

### Training the classification model

A five-fold cross-validation was adopted as mentioned before and the classifiers were trained using the training fold only. We adopted three classifiers to demonstrate the effectiveness of the chosen features. Logistic regression (Ng & Jordan, 2002), support vector machine (SVM) (Cortes & Vapnik, 1995), and random forest (RF) (Breiman, 2001), classifiers were used to distinguish between HGG and LGG images. The logistic classifier fits the distribution of data to the binomial distribution and provides a category related output with values between 0 and 1. The SVM is trained to maximize the margins of the plane separating the two categories in the feature space and is the most common classifier used for binary classification. RF is an ensemble classifier composed of a number of decision trees and it could lessen overfitting by training each decision tree using only a subset of the entire data. To train the logistic regression classifier, selected radiomics features were linearly regressed to binarized grades of glioma and then a radiomics score was constructed using a linear combination of regression coefficients and feature values. The score was fitted to the logistic model and a fixed threshold (=.5) was applied to distinguish between HGG and LGG. The cost function of the SVM was Lagrangian of the sum of the distance from each feature point to the marginal line. We chose a linear kernel for the SVM. The selected features from the feature selection step were vectorized and referred to as the 5D feature space. As a result, training of the SVM was performed using the 5D feature space. The RF model was also trained using the same information as the SVM and 200 decision trees were used for consensus result in the training step.

### Applying the model to the test cohort and statistics

We applied the trained models to the test cohort. The selected features and the associated coefficients from the training step were applied to the test cohort. The actual values of the features were computed from the test cohort. Performance of both cohorts was measured using the accuracy, sensitivity, specificity, and area under the curve (AUC) value of the receiver operating characteristic (ROC) curve. The positive case for the confusion matrix was set to HGG. The adjusted R-squared value and *p*-value were calculated to evaluate the degree of fit of the model. As we adopted a five-fold cross-validation, we repeated the procedures of feature selection, model training, and testing steps five times each time leaving a different test fold out. Each left out fold led to one set of performance measures, and thus we reported the average value of five measures. All analyses and evaluation procedures were performed using MATLAB (Mathworks Inc. Natick, MA, USA)

## Results

### Selected features from training

The top five features from the mRMR feature selection algorithm were chosen as significant radiomics features from each fold. Table 2 shows the most stable four radiomics features which were selected at least three times from the five-fold cross-validation. Regarding the category of features, the morphological property of tumor was most effective at discriminating HGG from LGG. Especially, spherical disproportion, which indicated how much the tumor shape was distorted from an ideal sphere, was found to be most valuable. The next most efficacious features were the GLCM features, which represent texture characteristic of the intra-tumoral area. Regarding the imaging modality, one was from T1 contrast enhanced image, and the other was from FLAIR. Regarding the type of ROIs, one was from ROI type I (non-enhancing tumor and necrotic region) and the other three were from ROI type II (enhancing, non-enhancing tumor and necrotic region).

**Table 2:** Selected features via mRMR based on stability over five folds.

	**Feature name**	**Modality**	**Category**	**ROI type**
1	Spherical Disproportion	Shape	Shape	1
2	Contrast	T1c	GLCM	2
3	Compactness	Shape	Shape	2
4	Autocorrelation	FLAIR	GLCM	2

### Model performance in the training step

**Figure 3:**
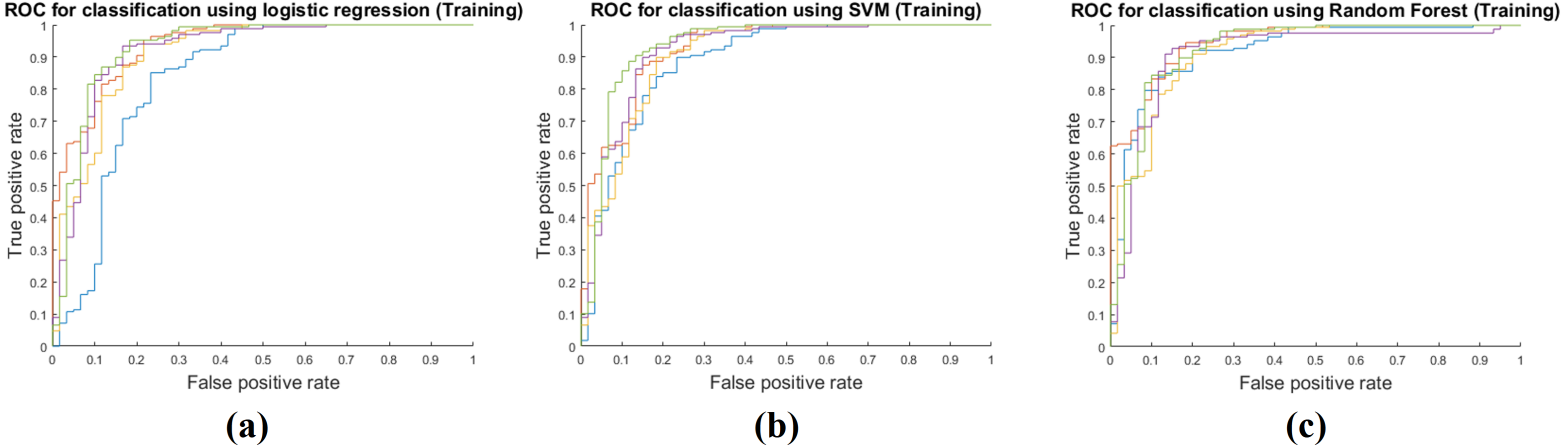
Performance curves of the five-fold cross validation in the training phase. (A) shows the ROC for the logistic regression classifier. (B) shows the ROC for the SVM classifier. (C) shows the ROC for the RF classifier.

Training performance of the three classifiers is shown in Table 3. Each performance value was calculated by averaging the five-fold cross validation results. The RF classifier showed the best training performance and three classifiers had an AUC of 0.9400 on average, which showed that the classifiers were very successful at modeling the training cohort. The accuracy, sensitivity, and specificity were measured as 0.9292, 0.9786, and 0.7911 on average. Figure 3 shows ROCs for the three classifiers for all five folds.

**Table 3:** Training performance measures using various classifiers.

**Classifier**	**Accuracy**	**Sensitivity**	**Specificity**	**AUC**	**AdjustedR-squared**	*p*-value
Logistic	0.8895	0.9643	0.6800	0.9066	0.4877	8.0686e−23
SVM	0.8983	0.9714	0.6933	0.9135	0.4461	6.5597e−13
RF	1	1	1	1	0.9537	7.4280e−148
Average	0.9292	0.9786	0.7911	0.9400		

**Notes.**

Each performance value was calculated by averaging the results of the five-fold cross validation.

SVM support vector machine RF random forest AUC area under the curve

### Model performance in test step

**Figure 4:**
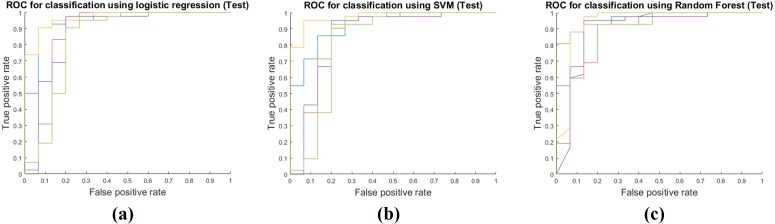
Performance curves of the five-fold cross validation in the test phase. (A) shows the ROC for the logistic regression classifier. (B) shows the ROC for the SVM classifier. (C) shows the ROC for the RF classifier.

Table 4 shows the results of applying the model constructed at the training stage to the test cohort. These results were obtained by fixing the image features selected by mRMR and all the model parameters. The actual feature values were computed from the test cohort. Same as the training phase, the RF classifier had the best performance with AUC 0.9213, and the average AUC of the three classifiers was 0.9030. The other performance measures were obtained in the same manner as the training phase. The average accuracy, sensitivity, and specificity were 0.8854, 0.9508 and 0.7022, respectively. Figure 4 shows ROCs for the three classifiers in the test cohort for all five folds.

**Table 4:** Test performance measures using various classifiers.

**Classifier**	**Accuracy**	**Sensitivity**	**Specificity**	**AUC**	**AdjustedR-squared**	*p*-value
Logistic	0.8877	0.9619	0.6800	0.9010	0.4882	5.6693e−23
SVM	0.8807	0.9476	0.6933	0.8866	0.3989	4.2893e−05
RF	0.8877	0.9429	0.7333	0.9213	0.5725	2.4653e−10
Average	0.8854	0.9508	0.7022	0.9030		

**Notes.**

Each performance value was calculated by averaging the results of the five-fold cross validation.

SVM support vector machine RF random forest AUC area under the curve

## Discussion

A radiomics approach can compute high-dimensional features from *in vivo* imaging modalities, which in turn were used to differentiate between HGG and LGG in this study. Our radiomics approach was tested with three classifiers and we obtained a high average AUC value of 0.9030 in the test cohort. In particular, the RF classifier showed the best performance with AUC 0.9213 for the test cohort. We also tested to see if the ensemble of the three classifiers using majority voting could be better than individual classifiers (Table 5). The RF classifier was significantly better other classifiers and thus the ensemble procedure did not improve the overall performance. Our main contribution was to fully leverage available multi-modal imaging with various machine learning approaches to distinguish between LGG and HGG.

**Table 5:** Test performance measures using various classifiers.

**Classifier**	**Accuracy**	**Sensitivity**	**Specificity**	**AUC**	**AdjustedR-squared**	*p*-value
Logistic	0.8877	0.9619	0.6800	0.9010	0.4882	5.6693e–23
SVM	0.8807	0.9476	0.6933	0.8866	0.3989	4.2893e–05
RF	0.8877	0.9429	0.7333	0.9213	0.5725	2.4653e–10
Ensemble	0.8947	0.9571	0.7200	0.8765	0.5471	2.2992e–09

**Notes.**

Each performance value was calculated by averaging the results of the five-fold cross validation.

SVM support vector machine RF random forest Ensemble ensembled classifier of three classifier AUC area under the curve

Others also attempted to differentiate HGG from LGG. Law et al. used relative cerebral blood volume measurements and metabolite ratios from proton MR spectroscopy, which resulted in a sensitivity of 0.950 and a specificity of 0.575 in discrimination between HGG and LGG (Law et al., 2003). A recent study applied conventional image processing approaches to a small scale data of 42 patients’ processed perfusion MRI and achieved a sensitivity of 0.966, specificity of 0.812 and AUC of 0.95 (Togao et al., 2016). Zacharaki et al. adopted texture features similar as our approach to differentiate between LGG and HGG (Zacharaki et al., 2009). They achieved an accuracy of 0.878 and AUC of 0.896 using SVM based recursive feature elimination (SVM-RFE) with a leave-one-out cross validation. The leave-one-out cross validation portion of that study might lead to overfitting, while our study could reduce the overfitting issue with a five-fold cross validation. In addition, SVM-RFE approach is tailored for the SVM classifier and thus applying the SVM-RFE in conjunction with other classifiers could lead to performance degradation.

We found four significant features that were stable through the five-fold cross validation. They were spherical disproportion of ROI type I (non-enhancing tumor and necrotic region), contrast of GLCM of ROI type II (enhancing, non-enhancing tumor, and necrotic region) from T1-contrast enhanced images, compactness of ROI type II, and autocorrelation of GLCM of ROI type II from FLAIR images (Table 2). The spherical disproportion and compactness measure how much an ROI shape differs from a sphere. The former is a measure based on volume measurements, while the latter is based on surface measurements. For spherical disproportion, an ideal sphere has value one and the value increases as the shape differs from the sphere. For compactness, an ideal sphere has value 0.0531 (i.e.,1/6 *π*) and the value decreases as the shape differs from the sphere. Glioma shape is a well-known factor associated with malignancy, as irregular tumor shape is often associated with higher malignancy and poor prognosis (Claes, Idema & Wesseling, 2007). We found that the shape of both non-enhancing and enhancing portion of the tumor were important in determining the glioma grades. Another important predictor of tumor prognosis is intratumoral heterogeneity (McGranahan & Swanton, 2015). We found two significant texture features: the contrast and autocorrelation of GLCM. The texture features quantify textural information within the ROI and can reveal patterns of intensity heterogeneity. The contrast of GLCM measures the local intensity variation of GLCM and autocorrelation of GLCM measures the magnitude of the fineness and coarseness of textural patterns. These texture features have often been identified as significant in other radiomics studies (Tixier et al., 2011; Davnall et al., 2012; Ganeshan et al., 2013; Grove et al., 2015; Bowen et al., 2017). One feature was from ROI type I (non-enhancing tumor and necrotic region) and the other three were from ROI type II, which included ROI type I plus the enhancing compartment. This confirmed that we need to consider both tumor core and the enhanced portion to evaluate the tumor grading.

There are related studies of gliomas using machine learning approaches. Kickingereder et al. estimated the progression-free and overall survival of GBM patients using T1, T1-contrast enhanced and FLAIR images (Kickingereder et al., 2016). They used principal component analysis (PCA) to develop radiomics signatures from high-dimensional features. PCA is effective at reducing dimensionality, but its results are difficult to interpret. Itakura et al. computed quantitative features from T1 images of GBM patients and found phenotypic clusters associated with molecular pathway activity through consensus clustering (Itakura et al., 2015). The adopted clustering was effective at demonstrating the association between imaging features and degree of malignancy, but the results were still difficult to interpret. One study developed machine learning-based prognostic imaging biomarkers of GBM images using multi-modal imaging, similar to our study (Cui et al., 2016). They adopted the L1-norm regularization method to select significant imaging features and predict overall survival (Tibshirani, 1996). In summary, our study was designed to produce stable and interpretable results of radiomics analysis compared to existing ones.

Recently, a machine learning algorithm known as deep learning (DL) has become the go-to methodology to drastically enhance the performance of existing machine learning techniques (Lecun, Bengio & Hinton, 2015). DL approaches have shown promise in tumor grading, diagnosis and prognosis prediction (Ertosun & Rubin, 2015; Litjens et al., 2016; Lao et al., 2017; Li et al., 2017). DL approach does not require the researcher to specify a set of features a priori, but can implicitly learn the features relevant to the problem, and thus can be effective for radiomics research. DL requires many more training samples compared to conventional machine learning approaches and additional issues arise when fine-tuning many hyper-parameters. These issues are challenging and we plan to pursue DL approaches in the future.

Our study has several limitations. We used open source data originally designated for a segmentation challenge, so we could not control for all factors between LGG and HGG groups. This might have included bias in patient selection. Independent validation using data from another clinical site is missing. This might hinder the applicability of our approach to new data. There was a class imbalance between two classes. We thought that each class had enough samples for statistical modeling. Still, the class imbalance issue might be alleviated by minority class oversampling techniques. The ROIs were provided by the database and the reproducibility of the ROI was not verified. World Health Organization recently announced a new tumor classification system of the central nervous system (Louis et al., 2016). It breaks up the glioma into five grades considering not only histological information but also isocitrate dehydrogenase mutation and 1p/19q codeletion. Updated grading of gliomas was unavailable to us and thus we used the information of the traditional grading system. Future studies should consider grading information on the new grading system.

## Conclusions

In conclusion, we showed that glioma grades could be accurately determined by a combination of high dimensional imaging features, an advanced feature selection method and machine learning classifiers. We believe the algorithm presented in our study might contribute to high-throughput computer aided diagnosis system for gliomas.

##  Supplemental Information

10.7717/peerj.5982/supp-1 Figure S1 A representative example of the two morphological features

10.7717/peerj.5982/supp-2 Supplemental Information 1 Description of radiomics features

10.7717/peerj.5982/supp-3 Supplemental Information 2 Code used for analysis

## Additional Information and Declarations

### Data Availability

The following information was supplied regarding data availability: We considered data from the MICCAI Brain Tumor Segmentation 2017 Challenge (BraTS 2017): https://www.med.upenn.edu/sbia/brats2017.html.

## References

[ref-1] Aerts HJWL, Velazquez ER, Leijenaar RTH, Parmar C, Grossmann P, Cavalho S, Bussink J, Monshouwer R, Haibe-Kains B, Rietveld D, Hoebers F, Rietbergen MM, Leemans CR, Dekker A, Quackenbush J, Gillies RJ, Lambin P (2014). Decoding tumour phenotype by noninvasive imaging using a quantitative radiomics approach. Nature Communications.

[ref-2] Bakas S, Akbari H, Sotiras A, Bilello M, Rozycki M, Kirby JS, Freymann JB, Farahani K, Davatzikos C (2017a). Advancing the cancer genome atlas glioma MRI collections with expert segmentation labels and radiomic features. Scientific Data.

[ref-3] Bakas S, Akbari H, Sotiras A, Bilello M, Rozycki M, Kirby J, Freymann J, Farahani K, Davatzikos C (2017b). Segmentation labels and radiomic features for the pre-operative scans of the TCGA-LGG collection. The Cancer Imaging Archive.

[ref-4] Bakas S, Akbari H, Sotiras A, Bilello M, Rozycki M, Kirby J, Freymann J, Farahani K, Davatzikos C (2017c). The Cancer Imaging Archive.

[ref-5] Beig N, Patel J, Prasanna P, Hill V, Gupta A, Correa R, Bera K, Singh S, Partovi S, Varadan V, Ahluwalia M, Madabhushi A, Tiwari P (2018). Radiogenomic analysis of hypoxia pathway is predictive of overall survival in Glioblastoma. Scientific Reports.

[ref-6] Bowen SR, Yuh WTC, Hippe DS, Wu W, Partridge SC, Elias S, Jia G, Huang Z, Sandison GA, Nelson D, Knopp MV, Lo SS, Kinahan PE, Mayr NA (2017). Tumor radiomic heterogeneity: multiparametric functional imaging to characterize variability and predict response following cervical cancer radiation therapy. Journal of Magnetic Resonance Imaging.

[ref-7] Breiman L (2001). Random forests. Machine Learning.

[ref-8] Claes A, Idema AJ, Wesseling P (2007). Diffuse glioma growth: a guerilla war. Acta Neuropathologica.

[ref-9] Clark K, Vendt B, Smith K, Freymann J, Kirby J, Koppel P, Moore S, Phillips S, Maffitt D, Pringle M, Tarbox L, Prior F (2013). The cancer imaging archive (TCIA): maintaining and operating a public information repository. Journal of Digital Imaging.

[ref-10] Cortes C, Vapnik V (1995). Support-vector networks. Machine Learning.

[ref-11] Cui Y, Tha KK, Terasaka S, Yamaguchi S, Wang J, Kudo K, Xing L, Shirato H, Li R (2016). Prognostic imaging biomarkers in glioblastoma: development and independent validation on the basis of multiregion and quantitative analysis of MR images. Radiology.

[ref-12] Davnall F, Yip CSP, Ljungqvist G, Selmi M, Ng F, Sanghera B, Ganeshan B, Miles KA, Cook GJ, Goh V (2012). Assessment of tumor heterogeneity: an emerging imaging tool for clinical practice?. Insights into Imaging.

[ref-13] Ertosun MG, Rubin DL (2015). Automated grading of gliomas using deep learning in digital pathology images: a modular approach with ensemble of convolutional neural networks.

[ref-14] Ganeshan B, Goh V, Mandeville HC, Ng QS, Hoskin PJ, Miles KA (2013). Non-small cell lung cancer: histopathologic correlates for texture parameters at CT. Radiology.

[ref-15] Grove O, Berglund AE, Schabath MB, Aerts HJWL, Dekker A, Wang H, Rios Velazquez E, Lambin P, Gu Y, Balagurunathan Y, Eikman E, Gatenby RA, Eschrich S, Gillies RJ (2015). Quantitative computed tomographic descriptors associate tumor shape complexity and intratumor heterogeneity with prognosis in lung adenocarcinoma. PLOS ONE.

[ref-16] Gutman DA, Dunn WD, Grossmann P, Cooper LAD, Holder CA, Ligon KL, Alexander BM, Aerts HJWL (2015). Somatic mutations associated with MRI-derived volumetric features in glioblastoma. Neuroradiology.

[ref-17] Haralick RM, Shanmugam K, Dinstein I (1973). Textural features for image classification. IEEE Transactions on Systems, Man, and Cybernetics.

[ref-18] Itakura H, Achrol AS, Mitchell LA, Loya JJ, Liu T, Westbroek EM, Feroze AH, Rodriguez S, Echegaray S, Azad TD, Yeom KW, Napel S, Rubin DL, Chang SD, Harsh GR, Gevaert O (2015). Magnetic resonance image features identify glioblastoma phenotypic subtypes with distinct molecular pathway activities. Science Translational Medicine.

[ref-19] Kickingereder P, Götz M, Muschelli J, Wick A, Neuberger U, Shinohara RT, Sill M, Nowosielski M, Schlemmer HP, Radbruch A, Wick W, Bendszus M, Maier-Hein KH, Bonekamp D (2016). Large-scale radiomic profiling of recurrent glioblastoma identifies an imaging predictor for stratifying anti-angiogenic treatment response. Clinical Cancer Research.

[ref-20] Lao J, Chen Y, Li Z-C, Li Q, Zhang J, Liu J, Zhai G (2017). A deep learning-based radiomics model for prediction of survival in glioblastoma multiforme. Scientific Reports.

[ref-21] Law M, Yang S, Wang H, Babb JS, Johnson G, Cha S, Knopp EA, Zagzag D (2003). Glioma grading: sensitivity, specificity, and predictive values of perfusion MR imaging and proton MR spectroscopic imaging compared with conventional MR imaging. American Journal of Neuroradiology.

[ref-22] Lecun Y, Bengio Y, Hinton G (2015). Deep learning. Nature.

[ref-23] Lee J, Jain R, Khalil K, Griffith B, Bosca R, Rao G, Rao A (2016). Texture feature ratios from relative CBV maps of perfusion MRI are associated with patient survival in glioblastoma. American Journal of Neuroradiology.

[ref-24] Li H, Zhu Y, Burnside ES, Drukker K, Hoadley KA, Fan C, Conzen SD, Whitman GJ, Sutton EJ, Net JM, Ganott M, Huang E, Morris EA, Perou CM, Ji Y, Giger ML (2016). MR imaging radiomics signatures for predicting the risk of breast cancer recurrence as given by research versions of MammaPrint, Oncotype DX, and PAM50 gene assays. Radiology.

[ref-25] Li Z, Wang Y, Yu J, Guo Y, Cao W (2017). Deep Learning based Radiomics (DLR) and its usage in noninvasive IDH1 prediction for low grade glioma. Scientific Reports.

[ref-26] Litjens G, Sánchez CI, Timofeeva N, Hermsen M, Nagtegaal I, Kovacs I, Hulsbergen-Van De Kaa C, Bult P, Van Ginneken B, Van Der Laak J (2016). Deep learning as a tool for increased accuracy and efficiency of histopathological diagnosis. Scientific Reports.

[ref-27] Louis DN, Ohgaki H, Wiestler OD, Cavenee WK, Burger PC, Jouvet A, Scheithauer BW, Kleihues P (2007). The 2007 WHO classification of tumours of the central nervous system. Acta Neuropathologica.

[ref-28] Louis DN, Perry A, Reifenberger G, Von DeimlingA, Figarella D, Webster B, Hiroko KC, Wiestler OD, Kleihues P, Ellison DW (2016). The 2016 World Health Organization classification of tumors of the central nervous system: a summary. Acta Neuropathologica.

[ref-29] McGranahan N, Swanton C (2015). Biological and therapeutic impact of intratumor heterogeneity in cancer evolution. Cancer Cell.

[ref-30] Menze BH, Jakab A, Bauer S, Kalpathy-Cramer J, Farahani K, Kirby J, Burren Y, Porz N, Slotboom J, Wiest R, Lanczi L, Gerstner E, Weber MA, Arbel T, Avants BB, Ayache N, Buendia P, Collins DL, Cordier N, Corso JJ, Criminisi A, Das T, Delingette H, Demiralp Ç, Durst CR, Dojat M, Doyle S, Festa J, Forbes F, Geremia E, Glocker B, Golland P, Guo X, Hamamci A, Iftekharuddin KM, Jena R, John NM, Konukoglu E, Lashkari D, Mariz JA, Meier R, Pereira S, Precup D, Price SJ, Raviv TR, Reza SMS, Ryan M, Sarikaya D, Schwartz L, Shin HC, Shotton J, Silva CA, Sousa N, Subbanna NK, Szekely G, Taylor TJ, Thomas OM, Tustison NJ, Unal G, Vasseur F, Wintermark M, Ye DH, Zhao L, Zhao B, Zikic D, Prastawa M, Reyes M, Van Leemput K (2015). The multimodal brain tumor image segmentation benchmark (BRATS). IEEE Transactions on Medical Imaging.

[ref-31] Ng A, Jordan MI (2002). On generative vs. discriminative classifiers: a comparison of logistic regression and naive bayes. Advances in Neural Information Processing Systems.

[ref-32] Pedano N, Flanders AE, Scarpace L, Mikkelsen T, Eschbacher JM, Hermes B, Ostrom Q (2016). The Cancer Imaging Archive.

[ref-33] Peng H, Long F, Ding C (2005). Feature selection based on mutual information: criteria of max-dependency, max-relevance, and min-redundancy. IEEE Transactions on Pattern Analysis and Machine Intelligence.

[ref-34] Prasanna P, Patel J, Partovi S, Madabhushi A, Tiwari P (2016). Radiomic features from the peritumoral brain parenchyma on treatment-naive multi-parametric MR imaging predict long versus short-term survival in glioblastoma multiforme: preliminary findings. European Radiology.

[ref-35] Rohlfing T, Zahr NM, Sullivan EV, Pfefferbaum A (2010). The SRI24 multichannel atlas of normal adult human brain structure. Human Brain Mapping.

[ref-36] Ryu YJ, Choi SH, Park SJ, Yun TJ, Kim JH, Sohn CH (2014). Glioma: application of whole-tumor texture analysis of diffusion-weighted imaging for the evaluation of tumor heterogeneity. PLOS ONE.

[ref-37] Scarpace L, Mikkelsen T, Cha S, Rao S, Tekchandani S, Gutman D (2016). The Cancer Imaging Archive.

[ref-38] Tibshirani R (1996). Regression selection and shrinkage via the Lasso. Journal of the Royal Statistical Society B.

[ref-39] Tixier F, Le Rest CC, Hatt M, Albarghach N, Pradier O, Metges J-P, Corcos L, Visvikis D (2011). Intratumor heterogeneity characterized by textural features on baseline 18F-FDG PET images predicts response to concomitant radiochemotherapy in esophageal cancer. Journal of Nuclear Medicine.

[ref-40] Togao O, Hiwatashi A, Yamashita K, Kikuchi K, Mizoguchi M, Yoshimoto K, Suzuki SO, Iwaki T, Obara M, Van Cauteren M, Honda H (2016). Differentiation of high-grade and low-grade diffuse gliomas by intravoxel incoherent motion MR imaging. Neuro-Oncology.

[ref-41] Van Griethuysen JJM, Fedorov A, Parmar C, Hosny A, Aucoin N, Narayan V, Beets-Tan RGH, Fillion-Robin JC, Pieper S, Aerts HJWL (2017). Computational radiomics system to decode the radiographic phenotype. Cancer Research.

[ref-42] Wu W, Lamborn KR, Buckner JC, Novotny PJ, Chang SM, O’Fallon JR, Jaeckle KA, Prados MD (2010). Joint NCCTG and NABTC prognostic factors analysis for high-grade recurrent glioma. Neuro-Oncology.

[ref-43] Yip SSF, Aerts HJWL (2016). Applications and limitations of radiomics. Physics in Medicine and Biology.

[ref-44] Zacharaki EI, Wang S, Chawla S, Soo D (2009). Classification of brain tumor type and grade using MRI texture and shape in a machine learning scheme. Magnetic Resonance in Medicine.

[ref-45] Zhou M, Chaudhury B, Hall LO, Goldgof DB, Gillies RJ, Gatenby RA (2017a). Identifying spatial imaging biomarkers of glioblastoma multiforme for survival group prediction. Journal of Magnetic Resonance Imaging.

[ref-46] Zhou M, Scott J, Chaudhury B, Hall L, Goldgof D, Yeom KW, Iv M, Ou Y, Kalpathy-Cramer J, Napel S, Gillies R, Gevaert O, Gatenby R (2017b). Radiomics in brain tumor: image assessment, quantitative feature descriptors, and machine-learning approaches. American Journal of Neuroradiology.

[ref-47] Zinn PO, Singh SK, Kotrotsou A, Hassan I, Thomas G, Luedi MM, Elakkad A, Elshafeey N, Idris T, Mosley J, Gumin J, Fuller GN, DeGroot JF, Baladandayuthapani V, Sulman EP, Kumar AJ, Sawaya R, Lang FF, Piwnica-Worms D, Colen RR (2018). A co-clinical radiogenomic validation study-conserved magnetic resonance radiomic appearance of Periostin expressing Glioblastoma in patients and xenograft models. Clinical Cancer Research.

