# Supplementary figures and images for: Classification of the glioma grading using radiomics analysis
Source: PeerJ. 2018 Nov 22;6:e5982. doi: 10.7717/peerj.5982 (PMC6252243; doi:10.7717/peerj.5982)

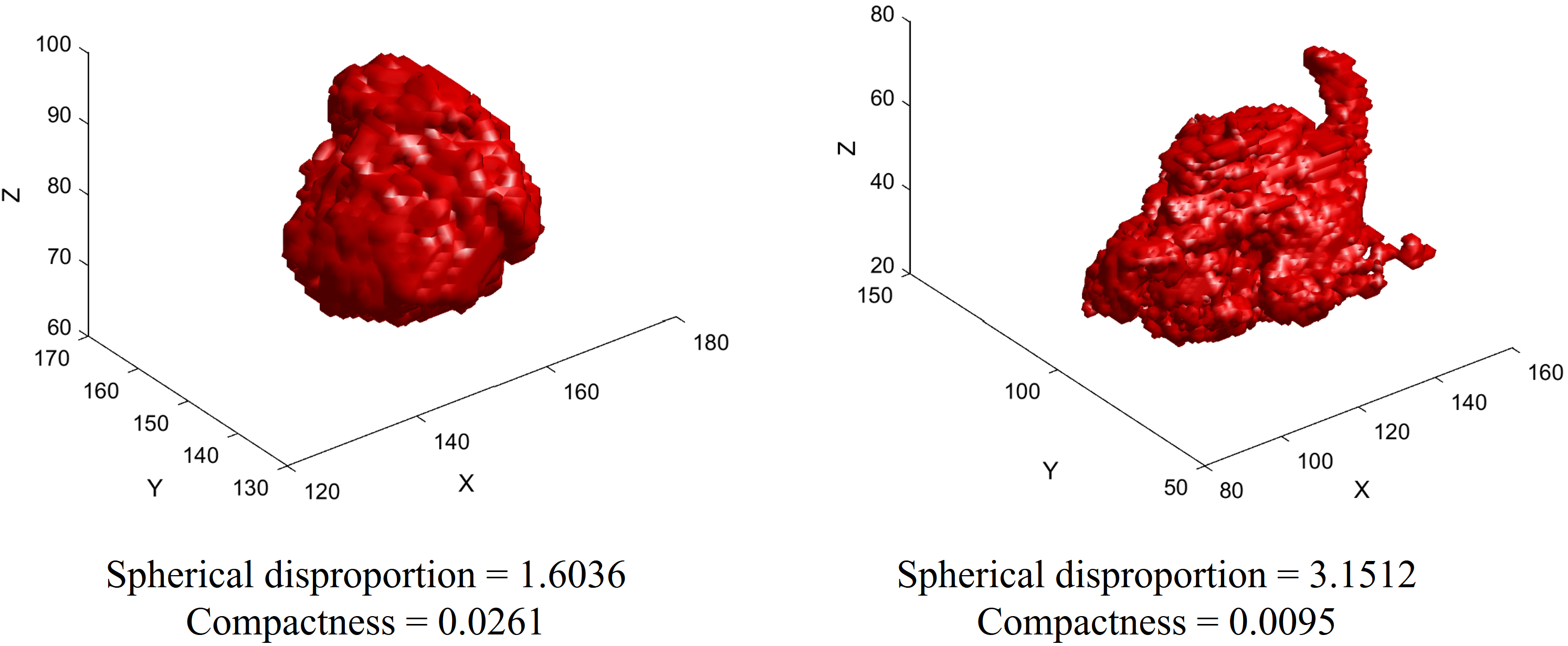

Supplement: Figure S1 [file peerj-06-5982-s001.png]
