# Supplementary material for: Classification of the glioma grading using radiomics analysis
Source: PeerJ. 2018 Nov 22;6:e5982. doi: 10.7717/peerj.5982 (PMC6252243; doi:10.7717/peerj.5982)
Supplement: Supplemental Information 1 [file peerj-06-5982-s002.docx]

Supplementary Information

Supplementary Table S1. description of radiomics features

| **Classes of texture features** | **Based methods** | **Parameter** | **Formula** | **Description** | |
| --- | --- | --- | --- | --- | --- |
| **Statistical based** | **1^st^ order features** (Histogram based features) | Maximum | $Max=max(X\left( i \right))$  Where $X$ denote the 3d image matrix | Measures maximum intensity value of a histogram | |
|  |  | Minimum | $Min=min(X\left( i \right))$  Where $X$ denote the 3d image matrix | Measures minimum intensity value of a histogram | |
|  |  | Median | $Median=\frac{X(i)}{2}$  Where $X$ denote the 3d image matrix | Measures median intensity value of a histogram | |
|  |  | Mean | $Mean=\frac{1}{N}\sum_{i}^{N} X(i)$  Where $X$ denote the 3d image matrix with $N$ voxel. | Measures mean intensity value of a histogram | |
|  |  | Variance | $Variance=\frac{1}{N-1}\sum_{i=1}^{N} \left( X\left( i \right)-\bar{x} \right)^{2}$ | Measures squared distances of each value of a histogram from the mean | |
|  |  | *Energy* | $Energy=\sum_{i}^{N} {X(i)}^{2}$  Where $X$ denote the 3d image matrix with $N$ voxel. | Measures squared magnitude value of a histogram | |
|  |  | Standard deviation | $Std=\left( \frac{1}{N-1}\sum_{i=1}^{N} \left( X\left( i \right)-\bar{x} \right)^{2} \right)^{1/2}$  Where $X$ denote the 3d image matrix with $N$ voxel. | Measures amount of variation of a histogram. | |
|  |  | Skewness | $Skewness=\frac{E{(x-\mu)}^{3}}{\sigma^{3}}$  Where $\mu$ is the mean of $x$, $\sigma$ is the standard deviation of $x$, $E$ is the expectation operator. | Measures asymmetry of a histogram. | |
|  |  | Kurtosis | $Kurtosis=\frac{{E(x-\mu)}^{4}}{\sigma^{4}}$  Where $\mu$ is the mean of$x$, $\sigma$ is the standard deviation of $x$, $E$ is the expectation operator. | Measures “peakedeness” of a histogram (flatness of histogram) | |
|  |  | Root mean square (RMS) | $RMS=\sqrt{\frac{1}{N}\sum_{n=1}^{N} \left\vert X_{n} \right\vert^{2}}$  Where $X$ denote the 3d image matrix with $N$ voxel. | Measures the square-root of the mean of the squares of the values of the histogram. This feature is another measure of the magnitude of a histogram | |
|  |  | Inter quartile range | $IQR=Q_{3}-Q_{1}$  Where $Q_{3}$ denote the 3^rd^ quartile of histogram, $Q_{1}$ denote the 1^st^ quartile of histogram | Measures of variability, based on dividing a histogram into quartiles | |
|  |  | Range | $Range=range(X\left( i \right))$ | Measures difference between the highest and lowest voxel values of a histogram | |
|  |  | Entropy | $Entropy=-\sum_{i=1}^{N_{l}} P(i)\log_{2} P(i)$  Where $P$ denote the first order histogram with $N_{l}$ discrete intensity levels. | Measures irregularity of a histogram. | |
|  |  | Uniformity | $Uniformity=\sum_{i=1}^{N_{l}} {P(i)}^{2}$  Where $P$ denote the first order histogram with $N_{l}$ discrete intensity levels. | Measures uniformity of a histogram. | |
|  |  | Percentile | $Percentile=\left( \frac{n^{th} percentile}{100} \right) X(i)$ | Measures intensity value at the 2.5^th^ , 25^th^ ,50^th^ ,75^th^ , and 97.5^th^ percentile on histogram | |
|  | **Higher order features**  (GLCM based features) | Autocorrelation | $Autocorrelation =\sum_{i=1}^{N_{g}} \sum_{j=1}^{N_{g}} \mathrm{ij}\mathbf{P}(i,j)$ | Measures of the magnitude of the fineness and coarseness of texture | |
|  |  | Cluster tendency | $Cluster tendency=$  $\sum_{i=1}^{N_{g}} \sum_{j=1}^{N_{g}} \left[ i+j-\mu_{x} -\mu_{y} \right]^{2}\mathbf{P}(i,j)$ | Measures of the homogeneity of GLCM | |
|  |  | Maximum probability | $Maximum probability=max\{P\left( i,j \right)\}$ | Measures maximum value of GLCM matrix | |
|  |  | Contrast | $Contrast=\sum_{i=1}^{N_{g}} \sum_{j=1}^{N_{g}} \left\vert i-j \right\vert^{2}\mathbf{P}(i,j)$ | Measures of the local intensity variation of GLCM | |
|  |  | Difference entropy | $Difference entropy=\sum_{i=0}^{N_{g}-1} \mathbf{P}_{x-y}(i)\log_{2} [P_{x-y}(i)]$ | Measures entropy of processed GLCM matrix Px-y | |
|  |  | Dissimilarity | $Dissimilarity=\sum_{i=1}^{N_{g}} \sum_{j=1}^{N_{g}} \left\vert i-j \right\vert\mathbf{P}(i,j)$ | Measures differences of entries in GLCM | |
|  |  | Energy | $Energy=\sum_{i=1}^{N_{g}} \sum_{j=1}^{N_{g}} \left[ \mathbf{P}\left( i,j \right) \right]^{2}$ | Measures of the homogeneity of GLCM | |
|  |  | Entropy | $Entropy=-\sum_{i=1}^{N_{g}} \sum_{j=1}^{N_{g}} \mathbf{P}(i,j)\log_{2} [\mathbf{P}\left( i,j \right)]$ | Measures irregularity of GLCM | |
|  |  | Homogeneity1 | $Homogeneity1=\sum_{i=1}^{N_{g}} \sum_{j=1}^{N_{g}} \frac{\mathbf{P}\left( i,j \right)}{1+\left\vert i-j \right\vert}$ | Measures closeness of GLCM | |
|  |  | Informational measure of correlation 1 (IMC1) | $IMC1=\frac{HXY-HXY1}{max\{HX,HY\}}$ | Secondary measure of Homogeneity1 | |
|  |  | Variance | $Variance=\sum_{i=1}^{N_{g}} \sum_{j=1}^{N_{g}} \left( i-\mu_{x} \right)^{2}P(i,j)$ | Measures dispersion of the parameter values around the mean of the combinations of reference and neighborhood pixels | |
|  |  | Sum average | $Sum average =\sum_{i=2}^{2N_{g}} \left[ iP_{x+y}\left( i \right) \right]$ | Measures the relationship between occurrences of pairs with lower and higher intensity values | |
|  |  | Sum entropy | $Sum entropy =-\sum_{i=2}^{2N_{g}} P_{x+y}(i)\log_{2} \left[ P_{x+y}\left( i \right) \right]$ | Sum of neighborhood intensity value differences | |
|  |  | Sum variance | $Sum variance=\sum_{i=2}^{2N_{g}} \left( i-SA \right)^{2}P_{x+y}(i)$ |  | |
|  |  | Inverse variance | $inverse variance=\sum_{i=1}^{N_{g}} \sum_{j=1}^{N_{g}} \frac{P(i,j)}{\left\vert i-j \right\vert^{2}}, i\neq j$ |  | |
|  |  | Inverse Difference Moment Normalized (IDMN) | $IDMN=\sum_{i=1}^{N_{g}} \sum_{j=1}^{N_{g}} \frac{P(i,j)}{1+\left( \frac{\left\vert i-j \right\vert^{2}}{N^{2}} \right)}$ | Measures the local homogeneity of an image | |
|  |  | Where $\mathbf{P}\left( i,j \right)$is the gray level co-occurrence matrix for ($\delta=1, \alpha=0)$,  $N_{g}$is the number of discrete intensity value in the image,  $N$ is the number of voxels in the ROI,  $\mu$ is the mean of $\mathbf{P}\left( i,j \right),$  $p_{x}\left( i \right)=\sum_{j=1}^{N_{g}} \mathbf{P}(i,j)$ is the marginal row probabilities,  $p_{y}\left( i \right)=\sum_{i=1}^{N_{g}} \mathbf{P}(i,j)$ is the marginal column probabilities,  $\mu_{x}$ is the expected value of marginal row probability,  $\mu_{y}$ is the expected value of marginal column probability,  $\sigma_{x}$ is the standard deviation of $p_{x}$,  $\sigma_{y}$ is the standard deviation of $p_{y}$,  $p_{x+y}\left( k \right)=\sum_{i=1}^{N_{g}} \sum_{j=1}^{N_{g}} \mathbf{P}\left( i,j \right) , i+j=k, k=2,3,\ldots,2N_{g}$,  $p_{x-y}\left( k \right)=\sum_{i=1}^{N_{g}} \sum_{j=1}^{N_{g}} \mathbf{P}\left( i,j \right) ,\left\vert i-j \right\vert=k, k=0,1,\ldots,N_{g}-1$,  $HX=-\sum_{i=1}^{N_{g}} \mathbf{P}_{x}(i)\log_{2} \left[ p_{x}(i) \right]$ is the entropy of $\mathbf{P}_{x}$,  $HY=-\sum_{i=1}^{N_{g}} \mathbf{P}_{y}(i)\log_{2} \left[ p_{y}(i) \right]$ is the entropy of $\mathbf{P}_{y}$,  $HXY=-\sum_{i=1}^{N_{g}} \sum_{j=1}^{N_{g}} \mathbf{P}\left( i,j \right)\log_{2} \left[ \mathbf{P}(i,j) \right]$is the entropy of $\mathbf{P}\left( i,j \right)$  $HXY1=-\sum_{i=1}^{N_{g}} \sum_{j=1}^{N_{g}} \mathbf{P}\left( i,j \right)\log(p_{x}\left( i \right)p_{y}\left( j \right))$. | | | |
|  | **Higher order features**  (ISZ based features) | Size-zone variability | $\frac{1}{\Theta}{\sum_{m=1}^{M} \left[ \sum_{n=1}^{N} \mathbf{P}\left( m,n \right) \right]}^{2}$ | | Variability in the size |
|  |  | Intensity variability | $\frac{1}{\Theta}{\sum_{n=1}^{N} \left[ \sum_{m=1}^{M} \mathbf{P}\left( m,n \right) \right]}^{2}$ | | Variability in the intensity |
|  |  | Where $\boldsymbol{P}\left( m,n \right)$ is the intensity size zone matrix  $\Theta$ represents the number of homogeneous areas in tumor,  $M$ is the number of distinct intensity values,  $N$ is the size of homogeneous area in the matrix $\boldsymbol{P}\left( m,n \right)$ | | | |
| **Morphological features** | **Shape and Size based features** | Compactness | $Compactness=\frac{V}{\sqrt{\pi}A^{\frac{3}{2}}}$  Where$V$ denote the volume and $A$ denote the surface area of the volume of interest (VOI) | | Quantifies how close an object to the smoothest shape, the circle |
|  |  | Surface area | $SA=\sum_{i=1}^{N} \frac{1}{2}\left\vert a_{i}b_{i}\times a_{i}c_{i} \right\vert$  Where $N$ is the total number triangle (coved surface area) and $a,b, c$ are edge vectors | | The surface area of the ROI |
|  |  | Convexity | $Convexity=\frac{V}{V^{'}}$  Where $V$ denote tumor volume and $V^{'}$ denote convex hull volume | | Measures ratio of the ROI volume contained within the tumor to the calculated convex hull volume |
|  |  | Sphericity | $Sphericity=\frac{36\pi\times{{(V}^{2})}^{\frac{1}{3}}}{A}$  Where $A$ denote area and $V$ denote tumor volume | | Measures of the roundness of the ROI |
|  |  | Maximum 3D diameter | See description in the next column | | Measures of the maximum 3D ROI diameter. It is measured as the largest pairwise Euclidean distance, between surface voxels of the ROI |
|  |  | Spherical disproportion | $Spherical disproportion=\frac{A}{4\pi R^{2}}$  Where $R$ is the radius of a sphere with the same volume as the ROI | | The ratio of the surface area of the ROI to the surface area of a sphere with the same volume as the ROI |
|  |  | Surface to volume ratio (SVR) | $SVR=\frac{A}{V}$  Where $A$ is area and $V$ is volume | | Surface to volume ratio |
|  | **Physical based features** | Volume | $Volume=R*number of voxels$  Where $R$ denote the 3d image resolution | | Volume of tumor (ROI) |
